# Effect of Transplanting Various Concentrations of a Composite of Human Umbilical Cord Blood-Derived Mesenchymal Stem Cells and Hyaluronic Acid Hydrogel on Articular Cartilage Repair in a Rabbit Model

**DOI:** 10.1371/journal.pone.0165446

**Published:** 2016-11-08

**Authors:** Yong-Beom Park, Chul-Won Ha, Jin-A Kim, Ji-Heon Rhim, Yong-Geun Park, Jun Young Chung, Han-Jun Lee

**Affiliations:** 1 Department of Orthopedic Surgery, Chung-Ang University Hospital, Chung-Ang University College of Medicine, 102 Heukseok-ro, Dongjak-gu, Seoul 06973, South Korea; 2 Department of Orthopaedic Surgery, Samsung Medical Center, Sungkyunkwan University School of Medicine, 81 Irwon-ro, Gangnam-gu, Seoul 06351, South Korea; 3 Stem Cell & Regenerative Medicine Research Institute, Samsung Medical Center, 81 Irwon-ro, Gangnam-gu, Seoul 06351, South Korea; 4 Department of Health Sciences and Technology, SAIHST, Sungkyunkwan University, Seoul, South Korea; 5 Department of Orthopedic Surgery, Jeju National University Hospital, Jeju National University School of Medicine, 15 Aran 13-gil, Jeju-si 63241, South Korea; 6 Department of Orthopaedic Surgery, Ajou University Hospital, Ajou University School of Medicine, 164 World cup-ro, Yeongtong-gu, Suwon 16499, South Korea; Instituto Butantan, BRAZIL

## Abstract

**Background:**

Mesenchymal stem cells (MSCs) are known to have therapeutic potential for cartilage repair. However, the optimal concentration of MSCs for cartilage repair remains unclear. Therefore, we aimed to explore the feasibility of cartilage repair by human umbilical cord blood-derived MSCs (hUCB-MSCs) and to determine the optimal concentrations of the MSCs in a rabbit model.

**Methods:**

Osteochondral defects were created in the trochlear groove of femur in 55 rabbits. Four experimental groups (11 rabbits/group) were treated by transplanting the composite of hUCB-MSCs and HA with various MSCs concentrations (0.1, 0.5, 1.0, and 1.5 x 10^7^ cells/ml). One control group was left untreated. At 4, 8, and 16 weeks post-transplantation, the degree of cartilage repair was evaluated grossly and histologically.

**Findings:**

Overall, transplanting hUCB-MSCs and HA hydrogel resulted in cartilage repair tissue with better quality than the control without transplantation (P = 0.015 in 0.1, P = 0.004 in 0.5, P = 0.004 in 1.0, P = 0.132 in 1.5 x 10^7^ cells/ml). Interestingly, high cell concentration of hUCB-MSCs (1.5×10^7^ cells/ml) was inferior to low cell concentrations (0.1, 0.5, and 1.0 x 10^7^ cells/ml) in cartilage repair (P = 0.394,P = 0.041, P = 0.699, respectively). The 0.5 x 10^7^ cells/ml group showed the highest cartilage repair score at 4, 8 and 16 weeks post transplantation, and followed by 0.1x10^7^ cells/ml group or 1.0 x 10^7^ cell/ml group.

**Conclusions:**

The results of this study suggest that transplantation of the composite of hUCB-MSCs and HA is beneficial for cartilage repair. In addition, this study shows that optimal MSC concentration needs to be determined for better cartilage repair.

## Introduction

Articular cartilage is known as a highly differentiated avascular tissue with low self-regeneration capacity [1]. Many researchers have attempted to increase the regeneration potential of damaged cartilage using cell-based therapies such as autologous chondrocyte implantation (ACI) [2]. Although autologous cells can be implanted without immune rejection, it has considerable limitations such as the invasiveness for cell harvest, long period of culture time, and difficulty in cell expansion. In addition, the biological activities of the cultured autologous cells are largely dependent on the age and genetic background of the patient, which may result in various therapeutic outcomes [3,4]. Therefore, mesenchymal stem cells (MSCs) with self-renewal and multi-lineage differentiation potential and hypo-immunogenic properties have been studied as an alternative option for cell therapy [5–7]. Most previous studies have investigated articular cartilage repair using MSCs from bone marrow [8–11]. The invasiveness in bone marrow collection, containing only a small percentage of MSCs, and a decreasing differentiation potential and number of MSCs with aging have limited their application [4]. Therefore, MSCs obtainable from other human tissues have been investigated [12–15]. In particular, human umbilical cord blood-derived MSCs (hUCB-MSCs) have emerged as an alternative for cell therapy because they have plentiful cell banking systems with non-invasive collection, immediate transplantation, and hypo-immunogenic properties [16,17]. In addition, hUCB-MSCs exhibit high proliferation potential and karyotype stability after prolonged expansion [18]. Several studies have demonstrated the chondrogenic differentiation potential of hUCB-MSCs in laboratory settings [19–22]. However, only a few studies have reported cartilage repair using hUCB-MSCs *in vivo* [23,24].

In addition, optimization of cell seeding concentration could be important for improving cartilage repair by cell-based therapies. Therefore, to improve cartilage repair by MSCs therapy, determination of crucial parameters such as appropriate delivery vehicle and optimal cell concentration should also need to be investigated. Hyaluronic acid (HA) hydrogel has been reported to be a suitable delivery vehicle for hUCB-MSCs in cartilage repair [23,24]. However, the optimal cell concentrations of hUCB-MSCs in the HA hydrogel for cartilage repair have not been fully investigated. Although several studies have demonstrated the effect of cell concentration on cell proliferation rate, to the best of our knowledge, only a couple of *in vivo* studies have reported the effect of MSC concentrations on cartilage repair [25,26]. Therefore, the objectives of this study were: 1) to explore the feasibility of transplanting hUCB-MSCs and HA hydrogel composites to repair articular cartilage defects in a rabbit model; and 2) to determine the optimal hUCB-MSCs concentrations for cartilage repair. We hypothesized that transplanting hUCB-MSCs and a HA hydrogel composite would produce significantly better results compared to non-transplantation. We also hypothesized that cartilage repair with high concentration of hUCB-MSCs would have better results than those with low concentration.

## Materials and Methods

### Isolation and harvest of hUCB-MSCs

hUCB was collected from umbilical veins after neonatal delivery through an independent cord blood bank with informed consent from pregnant mothers. The isolation and cultivation of MSCs were performed according to a previously published method [27]. Briefly, mononuclear cells were isolated by density gradient centrifugation at 550 x *g* for 30 minutes using Ficoll Hypaque (density 1.077 g/ml, Sigma, St. Louis, MO, USA). The separated mononuclear cells were then cultured in α-minimum essential medium (α-MEM, Gibco BRL, Carlsbad, CA, USA) supplemented with 15% fetal bovine serum (FBS, HyClone, Logan, UT, USA) at 37°C in a humidified atmosphere with 5% CO_2_. The culture medium as changed twice a week. After culturing for approximately two weeks, fibroblast-like adherent cells were observed. When the monolayer of MSC colonies reached 80% confluence, cells were trypsinized (0.25% trypsin, HyClone), washed, and resuspended in culture medium (α-MEM supplemented with 10% FBS, 1% antibiotics). The cells expressed CD105 and CD73, but did not express CD34, CD45, CD14, and HLA-DR as published previously regarding the surface markers of mesenchymal stem cells [27].

### Animals

Fifty-five healthy New Zealand White male rabbits between 7 and 8 months-old (weight, 3.0–3.5Kg, Orient Bio, Inc. Korea)were used (n = 11 per group). Animals were maintained in cages in a room with 12-hour day/night cycles, an ambient temperature of 20~26°C, a relative humidity of 30~70% and ad libitum access to water and a standard laboratory pellet diet. Animal selection and management, surgical protocol, and preparation followed protocols approved by the Institutional Animal Care and Use Committee of our institution. This study also followed the Institute of Laboratory Animal Resources guide in management and use the experimental animal with the Certification of Accreditation of Association for Assessment and Accreditation of Laboratory Animal Care International.

### Experimental protocol

Animals were divided into five study groups, including one control group and four experimental groups with various hUCB-MSC concentrations (0.1, 0.5, 1.0, or 1.5 x 10^7^ cells/ml, Table 1). Anesthesia was induced by inhalation of 5% Ether followed by intramuscular injection of a combination of Xylazine 5 mg/kg and Ketamine 35 mg/kg. Anesthesia was maintained with isoflurane. All surgical procedures were performed with all efforts to minimize suffering according to the protocol approved by the Institutional Animal Care and Use Committee. After cleaning with 10% Betadine solution, both knee joints of each rabbit were sterilely draped and opened using a medial parapatellar approach. The patella was laterally dislocated and osteochondral defects (3 mm in diameter with 3 mm in depth) were created in the trochlear groove by carefully drilling in vertical direction. After removing cartilage and bone debris, the boundaries of the drill hole were trimmed using a surgical knife and washed out. A mixture of hUCB-MSCs of different cell concentrations and 4% HA (Hyal 2000^®^, LG Life Science, South Korea) hydrogel was then transplanted into the area of the defect in each experimental group. The osteochondral defects in the control group (group 1) were left untreated. As there have been studies showing significant differences for cartilage repair between HA only and MSCs seeded HA, but no difference between defect only and HA only [23,28,29], we chose the defect only as the control group in this study. Following transplantation, the patellar retinaculum and overlying soft tissues were closed in layers. Rabbits were then allowed to move knee joints freely in the cages without any immobilization device. An intramuscular injection of antibiotics (Amikacin 12.5 mg/kg) was given immediately after the transplantation and once daily for 5 days afterwards. Intramuscular injections of a pain-killer (ketoprofen 4mg/kg) were given for the first 3 days. During the experiment, body weight changes, external physical appearance, behavioral changes and physiological changes were carefully observed. At 4, 8, and 16 weeks post-transplantation, rabbits were sacrificed for the evaluation of cartilage repair using a combination of Xylazine 10mg/kg and Ketamine 70mg/kg by intramuscular injection followed by a KCL 2ml/kg by intravenous injection. The rabbits were to be euthanized if they reached the humane endpoints such as body weight loss > 20%, loss of ability to ambulate (inability to access food or water), behavioral changes (vocalization, self mutilation, restless or still), provoked behavior (violently, or very weak and precomatose), significant physiological changes (body temperature ± 2°C, cardiac and respiratory rate change > 50%), presence of labored respiration, or presence of significant pain, swelling, redness or discharge from surgical incisions. Five rabbits were euthanized within 2 weeks after surgery based on humane endpoints (3 rabbits due to excessive weight loss > 20%, 2 rabbits due to self mutilation on wound with significant discharge and dehiscence of the surgical wound).

**Table 1:** Study design of the control group and the four experimental groups.

Group	Study Duration	Dose (cells/ml)	Number of Animals
Control Group			
Group1	4 weeks	0	3
	8 weeks		3
	16 weeks		5
Experimental Groups			
Group2	4 weeks	0.1 x 10 ^7^	3
	8 weeks		3
	16 weeks		5
Group3	4 weeks	0.5 x 10 ^7^	3
	8 weeks		3
	16 weeks		5
Group4	4 weeks	1.0 x 10 ^7^	3
	8 weeks		3
	16 weeks		5
Group5	4 weeks	1.5 x 10 ^7^	3
	8 weeks		3
	16 weeks		5

### Macroscopic evaluation

After being sacrificed, each rabbit was placed on an operating table and shaved around the knee joint area. Arthrotomy was performed to re-inspect the intra-articular structure. Any abnormal findings suggesting rejection or infection such as severe inflammation or extensive fibrosis were carefully examined. The degree of articular cartilage repair (including the degree of defect repair, integration to border zone, and macroscopic appearance) was assessed using the International Cartilage Repair Society (ICRS) macroscopic evaluation system [30].

### Microscopic evaluation

For histological analysis, hematoxylin and eosin (H & E) staining, Safranin-O staining, Masson’s Trichrome staining, and immunohistochemical staining for Type-II collagen were performed according to manuals provided by the manufacturers. Full-thickness samples (cartilage and bone) were taken from each group at 4, 8, and 16 weeks after transplantation. They were fixed in 10% formaldehyde, decalcified in 10% nitric acid for 3 days, dehydrated in graded ethanol, and embedded in paraffin wax. Paraffin-embedded sections (4 μm in thickness) were cut and deparaffinized. For H&E staining, sections were stained with Mayer's hematoxylin and counter-stained with eosin (DAKO Denmark A/S, Glostrup, Denmark). For detecting cartilage matrix production, sections were deparaffinized and hydrated in distilled water, stained with a 0.1% safranin-O solution (Sigma-Aldrich, St. Louis, MO, USA) for 5 min, dehydrated and cleared with 95% ethyl alcohol, absolute ethyl alcohol, and xylene. Collagen deposition and interstitial fibrosis were evaluated using Masson’s trichrome stain. Briefly, tissue sections were stained in Masson’s composition solution for 5 min and differentiated in 5% phosphotungstic acid for 10 min. Tissue sections were then stained in Aniline blue solution for 5 min. Excess stain was rinsed from slides with 0.2% acetic acid. Type II collagen within the cartilage was evaluated by immunohistochemistry. Sections were incubated with anti type-II collagen monoclonal antibody (Millipore Corporate, Billerica, MA, USA) at 4°C overnight and the reactivity was detected using a diaminobenzidine tetrahydrochloride substrate after incubation with an HRP-linked secondary antibody. All samples were mounted onto coverslips with Shandon* Xylene Substitute (Thermo Fisher Scientific Inc., Waltham, MA, USA). Images of stained sections were recorded using a light microscope (model Nikon Eclipse 600; Nikon Corp., Tokyo, Japan) fitted with a digital camera (Nikon DXM1200F).

To assess the histological cartilage repair efficacy, sections were analyzed using a modified O’Driscoll score [31]. The nature of the predominant tissue (cellular morphology and safranin O staining of matrix), structural characteristics (surface regularity, structural integrity, thickness, bonding to the adjacent cartilage), freedom from cellular changes of degeneration (hypocellularity, chondrocyte clustering), and freedom from degenerative changes in adjacent cartilage were assessed. All samples were evaluated independently by two observers who were blinded to sample information (such as belonging to which group).

### Statistical analysis

The scores generated for each group were analyzed with Kruskal-Wallis test. After analyzing the five groups regarding their differences in macroscopic and histological evaluation, a post-hoc test was performed using Mann–Whitney test. The significance level was set at a P value of 0.05. All statistical analyses were performed using SAS 9.3 (SAS Institute, Cary, NC, USA). Dataset used in this study is given in S1 and S2 Datasets.

## Results

### Macroscopic findings

**Fig 1:**
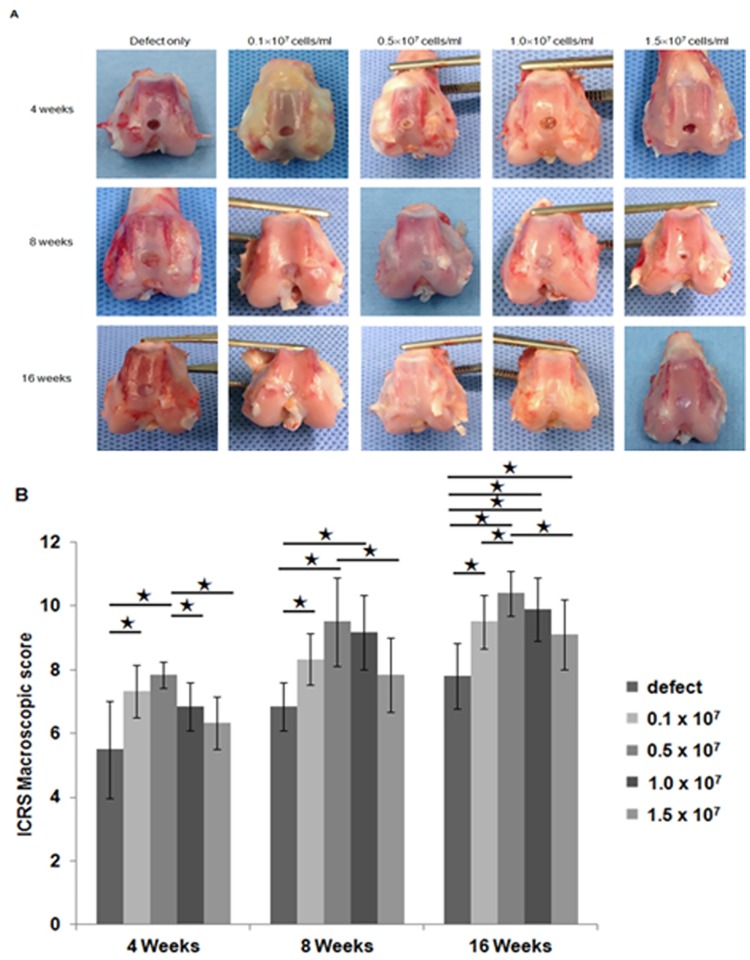
Gross appearance of repair tissue in osteochondral defects. (A) Gross appearance of the cartilage defect areas in the control group without transplantation and the experimental groups transplanted with different concentrations of hUCB-MSCs (0.1, 0.5, 1.0, or 1.5 x 10^7^ cells/ml) with hyaluronic acid hydrogel at 4 weeks, 8 weeks, and 16 weeks post transplantation. (B) International Cartilage Repair Society gross appearance scores for the control group and the four experimental groups at 4 weeks (n = 3/group), 8 weeks (n = 3/group), and 16 weeks (n = 5/group) post transplantation. (* p < 0.05, ** p<0.001).

At four weeks after transplantation, the articular surface of the defect site in the control group was irregular and more depressed than the surrounding normal cartilage. In addition, the defect site was filled with reddish white tissue clearly distinct from the normal articular cartilage. During the observation period between 8 and 16 weeks post transplantation, the defect sites in the control group (group 1) were still depressed compared to the surrounding native articular cartilage. The periphery of the defect was partially filled with white tissue with irregular surfaces (Fig 1A). In experimental groups (group 2 ~ group 5), the articular surfaces of the defect sites were relatively smoother. They showed similar coloration with the surrounding normal cartilage, unlike the control group. The border areas of the defect sites were less distinct and the depression levels were less significant compared to those in the control (Fig 1A). Overall, the experimental groups had significantly greater scores according to ICRS macroscopic evaluation system than that of the control group at each observation period (Fig 1B). When the four groups with different cell concentrations were compared, no significant difference in macroscopical findings was observed (Fig 1B).

### Microscopic findings

**Fig 2:**
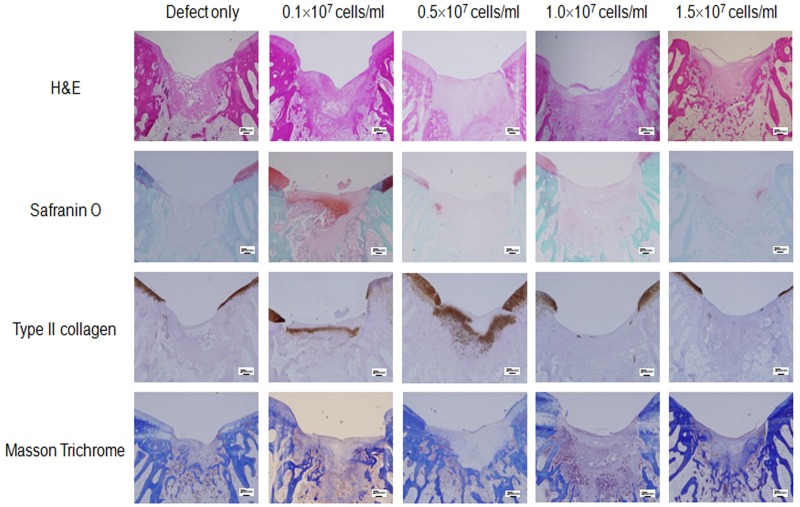
Histological analysis of the cartilage defect area at 4 weeks post transplantation. Representative microscopic features of the cartilage defect area in the control group (defect only) and the experimental groups (0.1 x 10^7^ cells/ml, 0.5 x 10^7^ cells/ml, 1.0 x 10^7^ cells/ml, or 1.5 x 10^7^ cells/ml) at 4 weeks post transplantation. Sectioned specimens were stained with H & E, safranin-O, Collagen type II antibody, and Masson’s Trichrome. × 40. Scale bars = 200 μm.

**Fig 3:**
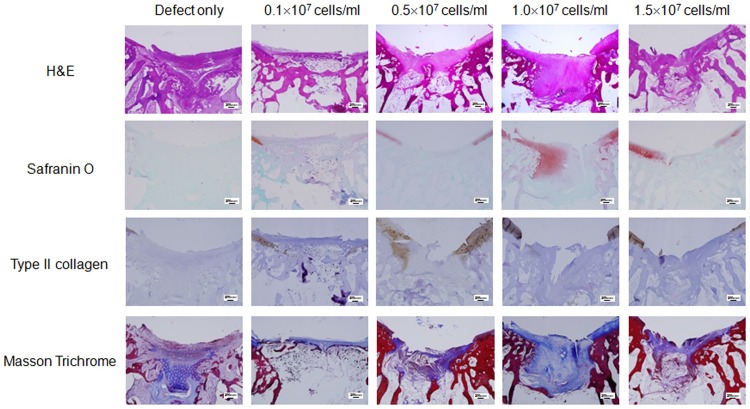
Histological analysis of the cartilage defect area at 8 weeks after transplantation. Representative microscopic features of the cartilage defect area in the control group (defect only) and experimental groups (0.1 x 10^7^ cells/ml, 0.5 x 10^7^ cells/ml, 1.0 x 10^7^ cells/ml, or 1.5 x 10^7^ cells/ml) at 8 weeks post transplantation. Sectioned specimens were stained with H & E, safranin-O, Collagen type II antibody, and Masson’s Trichrome. × 40. Scale bars = 200 μm.

**Fig 4:**
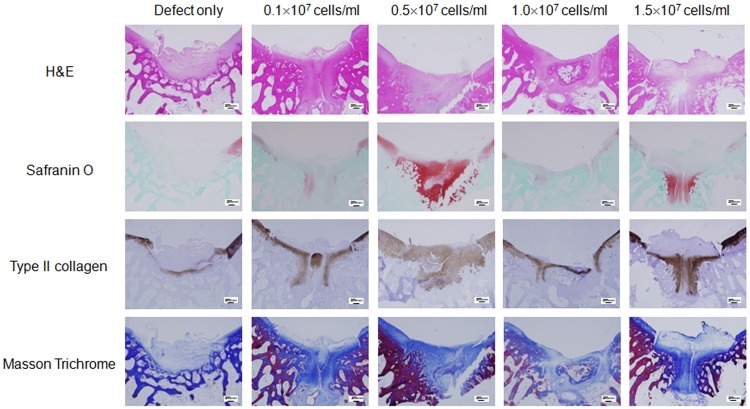
Histological analysis of the cartilage defect area at 16 weeks post transplantation. Representative microscopic features of the cartilage defect area in the control group (defect only) and experimental groups (0.1 x 10^7^ cells/ml, 0.5 x 10^7^ cells/ml, 1.0 x 10^7^ cells/ml, 1.5 x 10^7^ cells/ml) at 16 weeks post transplantation. Sectioned specimens were stained with H & E, safranin-O, Collagen type II antibody, and Masson’s Trichrome. × 40. Scale bars = 200 μm.

The defect sites in the control group were filled with more and more adipose and fibrotic cells in a time-dependent manner without findings of extracellular matrix (ECM) formation, suggesting poor cartilage repair in the control group (Figs 2–4, defect only). In contrast, the defect sites in the experimental groups were gradually replaced by hyaline cartilage-like tissue filling up to the height of the surrounding normal cartilage, although premature hyaline cartilage with immature chondrocytes was found in the early periods (between 4 and 8 weeks, Figs 2–4). In particular, restoration with hyaline-like cartilage seemed to occur more frequently in the deep zone than in the surface zone as shown by dense staining with safranin-O and immunostaining for type II collagen.

In detail, the H&E image in the control group showed cartilage loss in the defect site followed by replacement with mixed adipose and fibrotic tissue in the subchondral region. In the experimental groups, recovery of cartilaginous structure was observed in the defect site as indicated by pink color-stained tissue (Figs 2–4). Compared to pale safranin-O staining in the control group, the experimental groups showed strong orange to red colored stainings, indicating cartilage repair in the defect site. Also, denser staining was observed in the deep zone compared to that in the superficial zone, indicating more vigorous chondrogenesis in the deep zone. Compared to nearly pale staining in the defect area of the control group, the experimental groups showed evenly distributed and expanded staining, and indicated the presence of hyaline cartilage based on immunohistochemical staining for type II collagen (Figs 2–4). Masson trichrome staining showed pale blue staining in the control group but darker blue staining in the experimental groups, indicating presence of more collagen fiber distribution in the experimental groups (Figs 2–4).

**Fig 5:**
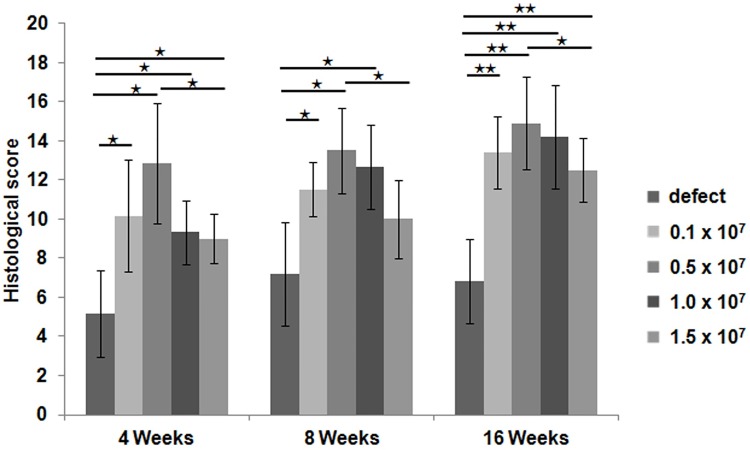
Histological score analysis. Using the modified O`Driscoll scoring scale, cartilage repair at the defect site was quantified in the control group (defect only) and the four experimental groups (0.1 x 10^7^ cells/ml, 0.5 x 10^7^ cells/ml, 1.0 x 10^7^ cells/ml, or 1.5 x 10^7^ cells/ml) at 4 weeks (n = 3/group), 8 weeks (n = 3/group), and 16 weeks (n = 5/group) post transplantation (maximum score, 24 points). (* p < 0.05, ** p < 0.001).

When the cartilage in each group was scored according to the modified O’Driscoll scoring system, the experimental groups were significantly superior to the control group (Fig 5). At 4 weeks post-transplantation, the 0.5 x 10^7^ cells/ml group showed the highest score, followed by the 0.1 x 10^7^ cells/ml group. At 8 weeks post-transplantation, the 0.5 x 10^7^ cells/ml group also showed the highest score, followed by the 1.0 x 10^7^ cells/ml group. At 16 weeks post-transplantation, the 0.5 x 10^7^ cells/ml group showed the highest score, followed by the 1.0 x 10^7^ cells/ml group. The 1.5 x 10^7^ cells/ml group showed the lowest score among the four experimental groups at 4, 8, and 16 weeks after transplantation.

## Discussion

In this study, we evaluated the effect of hUCB-MSCs at different concentrations with HA hydrogel on cartilage repair to determine the optimal cell concentration of hUCB-MSCs for cartilage repair. The results of the present study demonstrated that cartilage repair by transplanting hUCB-MSCs with HA hydrogel in the experimental groups was superior compared to the control group without transplantation based on macroscopic and histological examinations. In addition, our results revealed that the highest cell concentration of hUCB-MSCs did not increase the effect on cartilage repair. In fact, the highest cell concentration of hUCB-MSCs (1.5×10^7^ cells/ml) did not favor cartilage repair. Therefore, the concentration of hUCB-MSCs in HA hydrogel composite needs to be optimized to achieve a better cartilage repair. The results of the present study suggest that hUCB-MSCs with HA hydrogel could be used as a novel therapeutic option to treat articular cartilage defect *in vivo* when optimal cell concentration of hUCB-MSCs is used.

This study confirms the previous reports that the hUCB-MSCs can be safely and effectively used for cartilage repair without immunosuppression even in xenotransplantation model [23,24]. We believe that the immune modulatory function of the hUCB-MSCs reported in the literature should have led to this beneficial effect [32]. Regarding the possible mechanisms of the MSCs’ contribution to the repair of articular cartilage, there have been two possibilities suggested. One is by the differentiation potential of the transplanted MSCs, and the other is by the paracrine function of MSCs [22]. Yang et al. have suggested that secreted proteins can mediate interactions between human nucleus pulposus cells and MSCs [33]. In addition, several studies have reported that secreted proteins from mature human articular chondrocytes can suppress hypertrophy of MSCs during chondrogenesis [34–36]. These results suggest that specific interactions between hUCB-MSCs and subchondral progenitor cells initiated by paracrine action of the MSCs might be crucial for cartilage repair. However, the key mechanisms for cartilage repair with MSC-based therapy remain unknown.

An interesting finding observed in the present study is that high cell concentration resulted in unfavorable cartilage repair compared to low cell concentrations. All hUCB-MSCs concentrations resulted in favorable results in cartilage repair compared to the control group without hUCB-MSCs. Although lower hUCB-MSCs concentrations (0.1, 0.5, and 1.0 x 10^7^ cells/ml) seemed to be more effective for cartilage repair, higher concentration (1.5 x 10^7^ cells/ml) showed somewhat inferior cartilage repair capability. These findings are not consistent with results of previous *in vivo* studies [25,26]. One study with bone marrow-derived MSCs has reported that high cell seeding density can result in favorable cartilage repair in a rabbit model [26]. However, in that study, all of the three cell seeding densities (1 x 10^5^ cells/ml, 5 x 10^5^ cells/ml, and 1 x 10^6^ cells/ml) evaluated at 12 weeks post-transplantation were lower than the concentrations used in this study. They only found significant difference in histological scores between 1 x 10^5^ cells/ml and 1 x 10^6^ cells/ml. In another study with synovium-derived MSCs, the high cell density group (5 x 10^7^ cells/ml) showed statistically better results in histological score than the low cell density group (1 x 10^6^ cells/ml) at 4 weeks post-transplantation in a rabbit model [25]. The difference in the results between our study and this study might be due to differences in the characteristics of cell or the delivery vehicle. Therefore, optimal cell concentration of a specific MSC population for a certain therapeutic application seems to be determined.

There are two possible explanations for different results according to cell concentrations. First, there is still possibility that the viability of hUCB-MSCs might have been reduced due to excessively high concentration and aggregation of MSCs during transplantation [37]. Reduced viability of MSCs might have led to lower efficacy for cartilage repair. Therefore, optimal cell concentration of hUCB-MSCs to maintain viability during transplantation could be crucial for effective cartilage repair. Second, loss or damage of hUCB-MSCs by xenogenic immune rejection may result in less effective cartilage repair. Since UCB-MSCs and HA gel composite were transplanted onto the cartilage defect of rabbits in this study, there might be a chance of xenogenic immune rejection. Although there was no evidence of rejection observed in gross and histologic examinations, higher doses of transplanted hUCB-MSCs might have triggered more unexpected interactions with host immune cells. A few previous studies support the possibility of xenogenic immune reaction. De Bari et al. have reported immunorejection of human synovial MSCs in a immunocompetent mice model [38]. Osiecka-Iwan et al. have also demonstrated a strong humoral response in a rabbit after transplantation of rat chondrocytes [39]. Although undifferentiated hUCB-MSCs (and even a case of differentiated hUCB-MSCs) are hypo-immunogenic due to the lack of expression of histocompatibility complex (MHC) class II molecules and T-cell co-stimulatory factors with immunosuppressive properties *in vitro* [17,40–42], the immunogenicity of hUCB-MSCs has not been fully demonstrated *in vivo* yet. Therefore, under particular conditions, rejection of xenogenic hUCB-MSCs can occur to some extent, leading to decreased therapeutic effects of hUCB-MSCs. Based on these possibilities, the dose ranges of hUCB-MSCs for effective cartilage repair in this study provide important information in terms of identifying appropriate dose of hUCB-MSCs when considering human clinical trials of MSC-based therapy for cartilage defects.

This study has some limitations. First, xenogeneic MSCs transplantation is not a physiologic setting for clinical situation. Therefore, generalization of the results of this study might be difficult, especially for human beings. However, hUCB-MSCs have been demonstrated to have low immunogenicity with immunomodulatory activity [32]. Some *in vivo* studies using hUCB-MSCs have also shown no immune rejection [23,43]. Local inflammation, joint effusion, or unloading of the joint was not observed in this study or in previous studies with hUCB-MSCs for cartilage repair [23,24]. In those previous studies, consistent favorable results are observed for cartilage repair regardless of the species. Second, there was no control group using HA only in this study. As briefly mentioned in the Method section, some studies have shown that there are significant differences for cartilage repair between HA only and MSCs seeded HA, but no difference between defect only and HA only [28,29]. Another recent study has also shown no difference in cartilage repair between defect only and HA only in a rat model [23]. Also, in this study, we mainly focused to reveal the effect of variable concentrations of MSCs on the cartilage repair. Therefore, we chose the defect only model as a control group in this study.

In conclusion, this study showed that the application hUCB-MSCs with HA hydrogel composite to articular cartilage defects could be beneficial in cartilage repair. The effective dose ranges of transplanted hUCB-MSCs were also determined. Although further studies are needed to elucidate the precise underlying mechanisms contributing to the cartilage repair, our findings suggest that hUCB-MSCs HA hydrogel composite can be used for treating articular cartilage defects when optimal cell concentrations of hUCB-MSCs are used.

## Supporting Information

S1 Dataset Raw data of ICRS macroscopic score for macroscopic evaluation. (XLSX)

S2 Dataset Raw data of a modified O`Driscoll score for microscopic evaluation. (XLSX)
